# Spatial-temporal evolution of tuberculosis incidence rates in indigenous and non-indigenous people of Brazil, from 2011 to 2022

**DOI:** 10.1590/1980-549720230055

**Published:** 2023-12-11

**Authors:** Isabela Freitas Vaz, Natália Santana Paiva, Paulo Victor de Sousa Viana

**Affiliations:** I Fundação Oswaldo Cruz, Escola Nacional de Saúde Pública Sergio Arouca – Rio de Janeiro (RJ), Brazil.; II Universidade Federal do Rio de Janeiro, Instituto de Estudos em Saúde Coletiva – Rio de Janeiro (RJ), Brazil.; III Fundação Oswaldo Cruz, Escola Nacional de Saúde Pública Sergio Arouca, Centro de Referência Professor Hélio Fraga – Rio de Janeiro (RJ), Brazil.

**Keywords:** Indigenous peoples, Tuberculosis, Spatial analysis, Epidemiology, Incidence, Populações indígenas, Tuberculose, Análise espacial, Epidemiologia, Incidência

## Abstract

**Objective:**

To describe the space-time evolution of TB incidence rates (TI) in indigenous and non-indigenous people, according to the Federative Units (UF) of Brazil, from 2011 to 2022.

**Methods:**

Ecological, temporal, and spatial study on new tuberculosis cases in Brazil among indigenous and non-indigenous populations. Data from the Notifiable Diseases Information System (*Sinan*) were collected from 2011 to 2022 and stratified by Federal Unit, explored and statistically analyzed using R software version 4.2.3.

**Results:**

The mean TI among indigenous populations in Brazil was 71.7 new cases per 100,000 inhabitants, while for non-indigenous populations it was 28.6/100,000 inhabitants. The regions of the country that presented the highest (mean) incidence among indigenous populations were: Central-West (102.8/100,000 inhabitants), Southeast (99.6/100,000 inhabitants), and North (79.9/100,000 inhabitants). For non-indigenous populations the highest incidence was in the North region (36.5/100,000 inhabitants), followed by the Southeast (31.3/100,000), and the Northeast (27,4/100,000 inhabitants). The analysis showed that the highest incidence of TB cases among indigenous populations occurred in the states of: SP, RO, RJ, MS, MT e PA.

**Conclusion:**

High incidence of the disease compared to the non-indigenous population show the need for a specific approach to address the health needs of these populations. Regional disparities in incidence indicate the need to address socioeconomic and infrastructure issues that affect the health of indigenous populations.

## RESUMO

**Objetivo:**

Descrever a evolução espaço-temporal das taxas de incidência (TIs) de tuberculose (TB) em indígenas e não indígenas, segundo as unidades federativas do Brasil, no período de 2011 a 2022.

**Métodos:**

Estudo ecológico, temporal e espacial sobre os casos novos de tuberculose no Brasil em indígenas e não indígenas. Dados provenientes do Sistema de Informação de Agravos de Notificação (SINAN) foram coletados de 2011 a 2022 e estratificados por UF, analisados exploratória e estatisticamente por meio do *software* R 4.2.3.

**Resultados:**

A TI média entre indígenas no Brasil foi 71,7 casos novos para cada 100 mil habitantes, enquanto para não indígenas foi de 28,6/100 mil habitantes. As regiões do país que apresentaram as maiores incidências (médias) para indígenas foram: Centro-Oeste (102,8/100 mil hab.), Sudeste (99,6/100 mil hab.) e Norte (79,9/100 mil hab.), e para não indígenas foram: Norte (36,5/100 mil hab.), Sudeste (31,3/100 mil hab.) e Nordeste (27,4/100 mil hab.). A análise mostrou que a maior incidência de casos de TB nas populações indígenas ocorreu nos estados de São Paulo, Rondônia, Rio de Janeiro, Mato Grosso do Sul, Mato Grosso e Pará.

**Conclusão:**

Altas incidências da doença em comparação com a população não indígena mostraram a necessidade de abordagem específica para atender às necessidades de saúde dessas populações. As disparidades regionais nas incidências indicaram a necessidade de abordar questões socioeconômicas e de infraestrutura que afetam a saúde desses povos.

## INTRODUCTION

Tuberculosis (TB) continues to be an important public health problem worldwide — it is the 13^th^ overall cause of death and the second most common cause of infectious diseases after COVID-19 (above HIV/AIDS), even though it is a preventable, treatable, and curable disease. In 2021, around 10.6 million people worldwide fell ill from tuberculosis, resulting in approximately 1.6 million deaths^1^.

In response to this, the World Health Organization (WHO) set the goal of eliminating tuberculosis as a public health problem by 2035, for which it established challenging objectives, including early diagnosis of the disease in high-risk groups^2^. Among the high-risk groups, the indigenous population stands out as the one that also presents the highest incidences and prevalence of TB^3^. This situation can be explained by a combination of socioeconomic and health factors that affect this vulnerable group^4^.

TB is a historically serious public health problem in Brazil, ranking 18^th^ among the 30 countries responsible for 82% of the burden of the disease^1^. In 2022, even in a pandemic context, there was an increase in the number of new cases (78,087), representing an incidence rate of 36.3 cases per 100 thousand inhabitants. In the country, 1.1% of records occurred in the indigenous population^5^.

In Brazil, this population is one of the most vulnerable to TB due to factors such as low immunity, malnutrition, lack of access to health services and precarious housing^6^. Recent studies demonstrate that TB rates in indigenous peoples are up to four times higher than the national average^7–10^ recorded in the non-indigenous population.

According to Basta and Viana^11^, the risk of becoming ill with TB in indigenous populations is associated with the high prevalence of diseases, illnesses, and conditions considered risk factors for TB. Examples of these factors are the use of illicit drugs, food insecurity, and smoking. This finding raises the discussion about the importance not only of strategies to control TB, but also of social protection and poverty reduction actions.

In this context, this study aimed to describe the spatial-temporal evolution of TB incidence rates in indigenous and non-indigenous people, according to Brazilian states, from 2011 to 2022. This is an unprecedented study, which allows for in-depth knowledge about the national scope of the TB profile in indigenous and non-indigenous people, proposing a comparison of the crude incidence rates of the disease in this population.

## METHODS

This is an ecological study of spatial and temporal analysis, with a quantitative approach and a descriptive and exploratory nature, which analyzed tuberculosis incidence rate (IR) in the indigenous and non-indigenous population from 2011 to 2022 in the Brazilian states.

Data regarding new confirmed cases of tuberculosis, according to the year of treatment onset, during the study period, were collected from the Disease Information and Notification System (*Sistema de Informações de Agravos e Notificação* – SINAN), on the website of the Information Technology Department of the Brazilian Unified Health System (*Departamento de informática do Sistema Único de Saúde do Brasil* – DATASUS), while population data were obtained from the 2000 and 2010 Censuses of the Brazilian Institute of Geography and Statistics (*Instituto Brasileiro de Geografia e Estatística* – IBGE), available on official and public domain websites.

All reported new cases of TB, in which people declared themselves as white, black, brown or yellow are considered “non-indigenous”, according to the categories standardized by IBGE. New cases reported in SINAN with the categories “Ignored” or “Not completed” in the race/color variable were excluded from the study, as well as cases with the variable “Outcome” recorded as “Different diagnosis”.

TB IRs were estimated by race/color, year, and location using the following formula: IR = number of new cases of indigenous or non-indigenous TB, location and period, divided by the total population at risk of the same race/color, location and period multiplied by 100 thousand inhabitants.


$$\text{IR}=\frac{New\;cases\;of\;TB\;reported\;in\;a\;location,\;period,\;and\;race/color}{Residing\text{​}population\;at\;risk\;in\;the\;same\;location,\;period,\;and\;race/color}\times 100,000\;\text{inhab}\text{.}$$

Given the lack of intercensal estimates covering the different race/color categories for the locations and years under study, we used population projections to estimate the total population at risk (Supplementary material 1). These projections were based on population data from the 2000 and 2010 demographic censuses^12,13^.

The average annual growth rates through geometric extrapolation for the period from 2011 to 2022 were considered (for each race/color group and location). This extrapolation was based on data from the national demographic census conducted by IBGE in 2000 and 2010. We used the formula given by:


$$P_{t}=P_{0}e^{k_{g}\left(t-t_{0}\right)}$$

Where kg = *lnP*2-*lnP*0/*t*2-*t*0, t0 = 2000, t2 = 2010, P0, P2 = populations in years t0, t2, respectively, Pt = estimated population in years t = 2011, 2013, […], 2020, 2022.

The Empirical Bayesian (EB) method was used to reduce variations in unprocessed IRs, which occur in states with null rates or small populations (less than 20 thousand inhabitants). This method considered both the specific IRs for each state and the overall mean, applying proportional weights to the underlying populations at risk. To evaluate possible clusters or significant correlations of rates in the regions of the map, the Moran's I was calculated based on the unprocessed IRs.

The analyses of raw IR (by race/color group) were presented using a chart for Brazil, macro-regions, and states, calculating the mean (±standard deviation), minimum and maximum rates (considering all years of the study). To describe the spatio-temporal evolution of TB in Brazil, according to the state, in indigenous and non-indigenous populations, thematic maps of the distribution of TB incidences were created for each four-year period (2011–2014, 2015–2018, and 2019–2022), classifying the values based on natural breaks (Jenks) with five classes.

The exploratory and statistical analyses of this work were implemented in the free environment R 4.2.3 (R Development Core Team, 2023) and the data were structured in electronic spreadsheets in Microsoft Excel 2019 (Microsoft Corp., Redmond, WA, USA).

The secondary data used are of public and unrestricted access and were analyzed in aggregate, without identifying individuals, thus approval by CEP/CONEP was not necessary.

## RESULTS

During the study period (from 2011 to 2022), 765,768 new cases of TB were reported, 8,131 (1.1%) in self-declared indigenous individuals and 707,064 (92.3%) in non-indigenous individuals, according to the year of treatment onset. 50,573 (6.6%) notifications were excluded from the study in which the race/color variable was recorded as “ignored/blank”, due to the impossibility of defining the race/color.

The mean incidence of the disease in indigenous people in Brazil was 71.7/100,000 inhabitants, while for non-indigenous people it was 28.6/100,000 inhabitants. The regions that presented the highest incidences (means) for indigenous people were: Central West (102.8/100,000 inhabitants), Southeast (99.6/100,000 inhabitants), and North (79.9/100,000 inhabitants). For non-indigenous people, the highest mean incidences occurred in the North (36.5/100,000 inhabitants), in the Southeast (31.3/100,000 inhabitants), and in the Northeast (27.4/100,000 inhabitants).

As for states, the highest incidences (averages) for indigenous people were observed in Rio de Janeiro (197.5/100,000 inhabitants), in Mato Grosso (181.1/100,000 inhabitants), and in São Paulo (148.5/100,000 inhabitants). For non-indigenous people, the highest incidences occurred in Amazonas (67.2/100,000 inhabitants), in Rio de Janeiro (55.3/100,000 inhabitants), and in Acre (46/100,000 inhabitants), as presented in Chart 1.

**Chart 1:** Incidence of tuberculosis (per 100,000 inhabitants) in indigenous and non-indigenous people — states, macro-regions and Brazil, from 2011 to 2022.

Region/State		Mean		Standard deviation		Minimum		Maximum	
		Indigenous	Non-indigenous	Indigenous	Non-indigenous	Indigenous	Non-indigenous	Indigenous	Non-indigenous
Central West		102.8	20.0	81.9	9.1	0.0	5.1	444.9	43.1
	Distrito Federal (DF)	25.3	11.4	25.6	3.3	0.0	5.1	86.1	16.1
	Goiás (GO)	50.9	12.0	41.7	1.2	0.0	8.6	147.5	13.2
	Mato Grosso (MT)	181.1	30.4	103.6	6.6	67.6	18.5	444.9	43.1
	Mato Grosso do Sul (MS)	123.9	27.7	46.6	6.2	60.4	20.1	246.5	42.7
Northeast		48.5	27.4	37.8	6.7	0.0	14.4	181.7	46.3
	Alagoas (AL)	28.1	25.3	15.3	4.3	0.0	16.3	55.7	32.5
	Bahia (BA)	35.1	24.2	12.3	5.1	12.1	15.0	57.3	34.9
	Ceará (CE)	50.4	33.0	22.0	4.9	20.3	20.7	100.0	41.3
	Maranhão (MA)	93.2	26.3	25.4	2.1	43.5	23.7	117.2	29.9
	Paraíba (PB)	9.4	22.5	8.2	4.2	0.0	14.4	23.3	28.0
	Pernambuco (PE)	26.1	40.3	6.2	4.5	13.8	30.8	36.0	46.3
	Piauí (PI)	67.2	19.8	52.3	3.0	22.5	15.6	162.6	26.4
	Rio Grande do Norte (RN)	94.9	27.7	45.7	3.4	38.7	21.7	181.7	33.8
	Sergipe (SE)	35.5	27.8	22.6	3.6	0.0	22.2	69.5	35.3
North		79.9	36.5	42.5	16.5	8.9	9.2	215.3	77.8
	Acre (AC)	86.3	46.0	30.0	4.5	31.3	38.0	136.9	54.9
	Amapá (AP)	48.1	27.5	30.9	5.2	8.9	19.3	109.0	39.2
	Amazonas (AM)	69.8	67.2	13.2	5.8	36.3	59.5	86.3	77.8
	Pará (PA)	145.4	38.9	40.4	5.5	86.1	27.0	215.3	47.3
	Rondônia (RO)	111.8	30.3	52.2	4.3	28.1	19.3	207.8	34.3
	Roraima (RR)	55.9	33.2	16.4	12.0	30.8	20.5	84.3	62.1
	Tocantins (TO)	53.6	10.3	20.1	1.0	22.0	9.2	77.0	12.8
Southeast		99.6	31.3	81.4	15.7	0.0	0.2	330.7	68.8
	Espírito Santo (ES)	20.0	24.3	21.2	10.4	0.0	0.8	64.9	34.7
	Minas Gerais (MG)	35.5	14.9	12.0	2.0	19.7	10.3	61.3	19.3
	Rio de Janeiro (RJ)	197.5	55.3	40.0	7.0	138.5	40.8	283.4	68.8
	São Paulo (SP)	148.5	32.1	82.9	9.9	0.0	0.2	330.7	38.7
South		39.6	25.3	19.7	8.5	10.9	0.1	111.0	40.8
	Paraná (PR)	34.6	18.2	12.9	2.4	18.9	11.8	65.0	21.8
	Rio Grande do Sul (RS)	57.9	34.6	30.8	10.8	14.3	0.1	111.0	40.8
	Santa Catarina (SC)	29.5	23.6	12.2	3.6	10.9	15.6	51.9	27.2
Brazil		71.7	28.6	17.6	5.6	39.0	16.8	89.3	34.0

For the indigenous population, the regions with the highest incidences were the Central West (444.9±81.9) and the Southeast (330.7±81.4). As for non-indigenous people, the highest incidences were observed in the North (77.8±16.5) and in the Southeast (68.8±15.7). When analyzing the states, the highest incidences among indigenous people were recorded in Mato Grosso (444.9±103.6), Rio de Janeiro (283.4±40), São Paulo (330.7±82.9), Pará (215.3±40.4), and Rondônia (207.8±52.2). Meanwhile, the non-indigenous population presented higher incidences in Amazonas (77.8±5.8), Rio de Janeiro (68.8±7), and Acre (54.9±4.5), as demonstrated in Chart 1.

**Figure 1:**
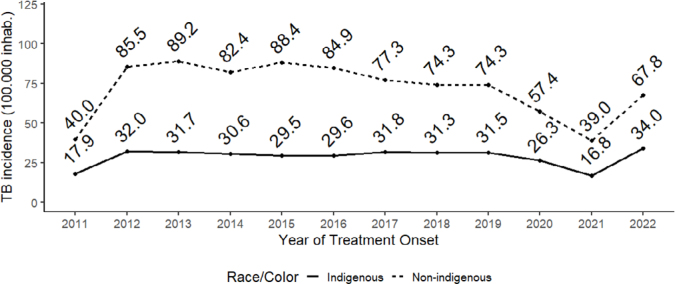
Time series of tuberculosis incidence rates in indigenous and non-indigenous populations. Brazil, 2011–2022.

Figure 1 shows that the indigenous population had the highest incidence in 2013 (89.2/100,000 inhabitants), while the lowest was recorded in 2021 (39/100,000 inhabitants). With regard to the non-indigenous population, it was found that the highest incidence occurred in 2012 (32/100,000 inhabitants) and the lowest in 2021 (16.8/100,000 inhabitants). In both populations studied, there was an increase in rates in 2012 compared to 2011, as well as in 2022 compared to 2021. This was followed by a sharp drop in TIs in 2020 and 2021.

**Figure 2:**
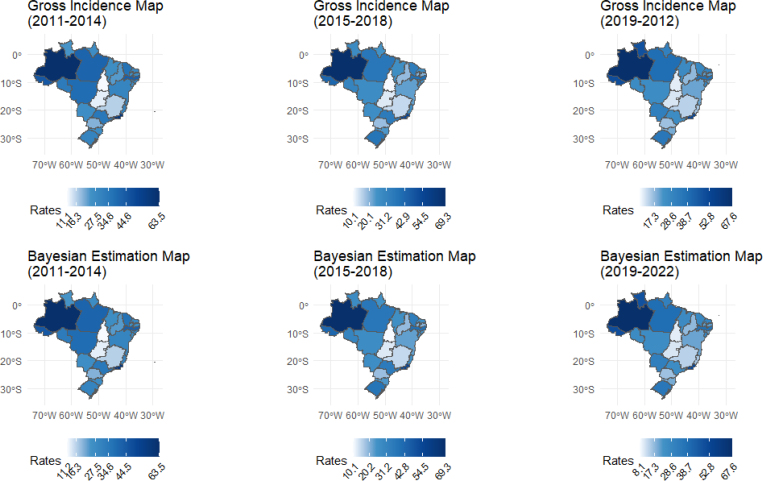
Tuberculosis incidence maps in non-indigenous people — Brazil, from 2011 to 2022, in four-year periods.

When analyzing the maps in Figure 2, one can see a certain uniformity in the spatial distribution of gross and adjusted IRs over the period. It is also evident that during the four-year periods, Amazonas and Rio de Janeiro recorded rates above 50/100,000 inhabitants. Furthermore, the high incidence rates sustained in Acre are worth highlighting, which remained above 40/100 thousand inhabitants. The application of the local smoothing method maintained this behavior for the mentioned states, due to the lower fluctuation of IRs for the non-indigenous population.

**Figure 3:**
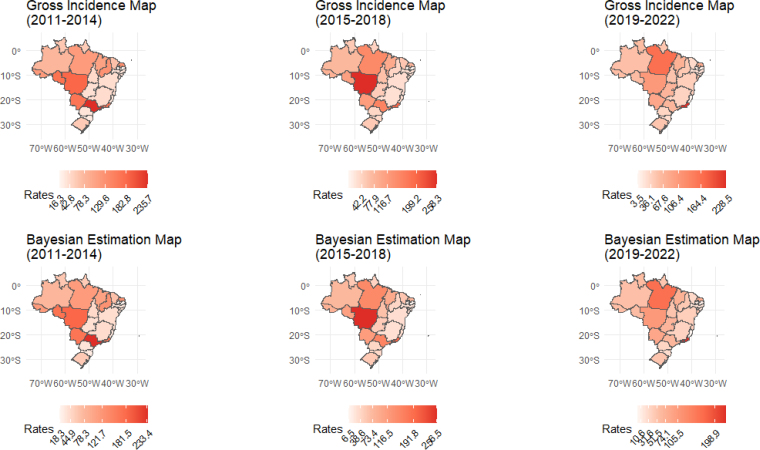
Maps of tuberculosis incidence in indigenous people — Brazil, from 2011 to 2022, in four-year periods.

Unlike the results found in the distribution of TB incidence in the non-indigenous population, the maps presented in Figure 3 revealed a more varied spatial distribution of IRs throughout the study period for the indigenous population. It was possible to identify high incidences during this period in states such as São Paulo, Rondônia, Rio de Janeiro, Mato Grosso do Sul, Mato Grosso, and Pará, with values above 150/100,000 inhabitants. A particularly high value, of 444.9/100,000 inhabitants, was observed in Mato Grosso, as indicated in Chart 1.

Analyzing the scales of the Bayesian estimates maps for the indigenous population, it was possible to identify subtle but notable changes in relation to the variations in IRs. The states that presented the most significant changes in their rates in the first four years were: Piauí, which registered a reduction from 126.2 to 120.5/100,000 inhabitants; Rio Grande do Norte, with a decrease from 129.6 to 121.7/100,000 inhabitants; Sergipe, with an increase from 34.8 to 41.3/100,000 inhabitants; and the Federal District, which increased from 17.7 to 24/100,000 inhabitants. In the second four-year period, Rio de Janeiro saw a drop from 199.2 to 191.8/100 thousand inhabitants. In the third four-year period, Rio de Janeiro again experienced a decrease, going from 228.5 to 198.9/100,000 inhabitants. During the period, the states with the lowest incidences for the indigenous population were Paraíba, Federal District, and Espírito Santo.

For the entire period of the study, a Moran index with a “high-high” classification was observed for the indigenous people of Amazonas and Bahia, while for non-indigenous people, a positive spatial correlation was identified in the states of Amazonas, Roraima, Acre, Rondônia, and Goiás (Supplementary material 2).

**Figure 4:**
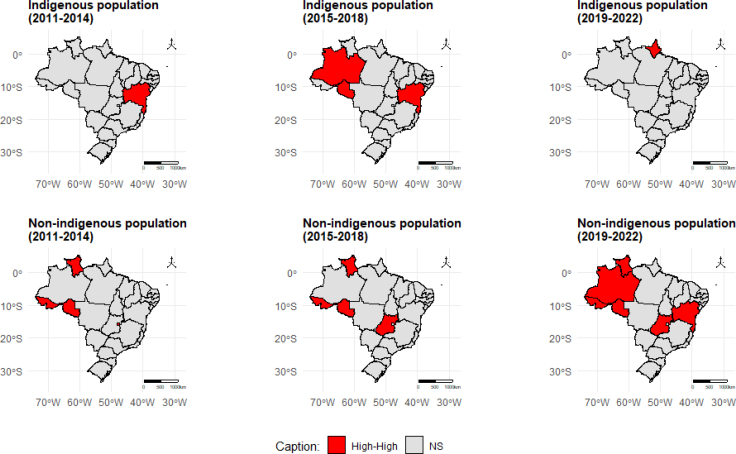
Spatial correlation of mean Bayesian incidence rates of tuberculosis in indigenous and non-indigenous people — Brazil, from 2011 to 2022, in four-year periods.

Considering the four-year periods for both populations, it was observed that, for the indigenous population, Bahia presented a high risk in the two consecutive four-year periods, while in the second, a high risk was observed in Amazonas and Rondônia. In the case of the non-indigenous population, a cluster was identified in the states of the Northern Region only in Rondônia, Acre, Roraima, and Amazonas, in the previous four years. In the last four-year period, another cluster was noticed, this time involving Bahia and Goiás (Figure 4).

## DISCUSSION

This is the first time that SINAN notification data has been used to investigate the burden of TB in indigenous and non-indigenous people in Brazil. This study enabled an overview of the distribution of the disease in the national territory over the last decade, considering the regions and their states. The results revealed that indigenous people are exposed to high incidences, presenting marked regional disparities.

In line with other studies on the disease based on race/color^7,8^, the results presented corroborate the high incidences in the Brazilian indigenous population, with the exception only of the states of Paraíba and Pernambuco, which presented higher rates for the non-indigenous population throughout the analyzed period. However, comparing IRs in the populations under study, it was concluded that, on average, IRs in indigenous peoples are often four times higher than those observed in non-indigenous people.

In the study on TB trends in indigenous people^10^, also at national level, a stable trend was identified, but with high values in the period from 2011 to 2017. The time series of this study corroborates the findings of the aforementioned survey, showing a gradual reduction in IRs from 2017 in Brazil.

The COVID-19 pandemic also had a significant impact on records of notifiable diseases^14–16^. In the case of TB, in the pandemic context, some interventions, such as active searches and preventive therapies, were postponed or no longer given priority. It is also important to consider that many new cases ended up being lost due to the similarity of the symptoms of tuberculosis and COVID-19, in addition to the reduction in demand for health services caused by the fear of infection by the new coronavirus and even the overload of health units^17–21^.

In Brazil, it was possible to observe this impact through the analysis of time series in all regions, showing lower notification of new cases and in IRs in 2020, with the exception only of the states mentioned in the results. In general, new TB cases are heterogeneously distributed across the country. Of the regions covered by the study, the Central West, Southeast, and North had the highest incidences for the indigenous population, while the North, Southeast, and Northeast stood out for the non-indigenous population.

Given the impact of the pandemic on notifications of new cases, sensitive and careful monitoring of tuberculosis epidemiological and operational indicators is recommended in order to evaluate disease control actions, contributing to reformulating and updating the National Plan to End Tuberculosis, aiming to meet the target of ten cases per group of 100,000 inhabitants by 2035.

When analyzing the states, it was observed that the non-indigenous population had higher rates in Amazonas, Rio de Janeiro, and Pará. This finding is in agreement with the information presented in the TB epidemiological bulletins from the Ministry of Health, from 2018 to 2022^22–24^, which highlight that Rio de Janeiro presented considerably high incidence and mortality rates compared to other states in the country.

This study also highlighted the high incidences of the disease in the indigenous population in São Paulo and Rio de Janeiro. These occurrences can be attributed both to the high demographic density and the configuration of population agglomeration in their urban centers, and to the gradual decrease in the population projection of indigenous people over time, which can affect the calculation of rates for more recent periods^10^.

The analysis of TB incidence in indigenous populations in Rondônia, conducted by Melo et al.^25^, also reveals a decrease in rates from 1997 to 2006. This fact is hypothetically related to the non-use of ethnicity as a surname for residents of the indigenous lands studied, concealing possible cases. During some periods of the study, it was reported that some municipalities and indigenous lands did not report any cases of the disease, raising the hypothesis of underreporting.

As shown in the results, the thematic maps reveal a dynamic distribution of TB in the indigenous population, characterized by a corridor with high incidence rates throughout the period of analysis, in the North, Central West, and Southeast regions. This concentration can be explained by the fact that a large portion of the indigenous population is located in the states of the Amazônia Legal^26^.

Analyzing the impact of TB on indigenous populations in Brazil presents significant challenges due to the extensive cultural diversity and particular forms of territorial distribution, with 729 indigenous lands recognized or in the process of being demarcated and 34 Special Indigenous Health Districts (*Distritos Sanitários Especiais Indígenas* – DSEIs). These factors result in geographic barriers, cultural limitations, including understanding the health-disease process and traditional indigenous medicine, as well as linguistic barriers, since Brazil is home to 305 ethnicities that speak 274 languages^27,28^.

These people have historically faced poor housing and sanitation conditions, as well as difficulties in accessing drinking water and food insecurity. These factors aggravate the risk of TB infection^29^, since, according to the WHO^1^, malnutrition is the main risk factor for developing the disease. Furthermore, barriers to accessing health services, such as lack of transportation and health professionals, along with socioeconomic issues, still represent challenges for diagnosing the disease and treating patients^30^.

The calculation of IRs presented limitations, since there was no standardization of rates in relation to age group and gender. Also, the population data used were obtained through intercensal periods, which may not accurately represent population growth of the regions and states of Brazil, which present different dynamics and configurations.

The findings of this study allowed us to conclude that, at a national level, there was a gradual reduction in mean IRs in the indigenous population from 2016 onward. However, TB continues to disproportionately affect indigenous peoples compared to non-indigenous ones, with considerably higher incidences to the expected results for 2020, as established by the National Plans to End Tuberculosis, from 2017 to 2020 and from 2021 to 2025^31,32^, which advocated a 20% reduction in the incidence of TB from 2015 to 2020.

It is important to highlight that Brazil has a history of social inequality that results in several social and health problems. Furthermore, it is necessary to understand the historical process of illness among indigenous peoples as a social phenomenon directly related to colonization and territorial invasions. The scenario of these populations becoming ill reflects the negative impact caused by the lack of guarantee of indigenous rights in the political context. In view of this, greater mobilization on the part of health and political authorities is suggested with a view to improving the quality of life and effective management of the legal framework and public health policies already implemented for this population.

This study highlights the need to adopt effective measures to combat the incidence of TB in the indigenous population in Brazil. The high rates of the disease in this group, compared to non-indigenous groups, indicate the need for a specific approach to meet their health demands.

The striking regional disparities in incidences demonstrated the importance of addressing socioeconomic and infrastructure issues that affect the health of these people, especially the lack of access to adequate health services.

Finally, it is essential to prioritize socioeconomic support and the implementation of public policies aimed at reducing social inequalities and guaranteeing indigenous populations access to quality health services.

The recent decree published in the Official Gazette of the Union^33^, far from being a definitive alternative, represents progress in the aforementioned issues, establishing the Interministerial Committee for the Elimination of Tuberculosis and Other Socially Determined Diseases (*Comitê Interministerial para a Eliminação da Tuberculose e de Outras Doenças Determinadas Socialmente* – CIEDS). In this context, the first technical production of CIEDS^34^ presents as a proposal for multisectoral action the expansion of access, early diagnosis, and timely treatment for indigenous peoples with the hope of contributing significantly to improving the quality of life of this population and effectively combating tuberculosis.
