# Supplementary material for: Spatial-temporal evolution of tuberculosis incidence rates in indigenous and non-indigenous people of Brazil, from 2011 to 2022
Source: Rev Bras Epidemiol. 2023 Dec 11;26:e230055. doi: 10.1590/1980-549720230055 (PMC10715319; doi:10.1590/1980-549720230055)
Supplement: Supplementary file 1 [file 1980-5497-rbepid-26-e230055-s1.pdf]

Material Suplementar 1 - Correlação espacial das TI bayesianas médias de TB em indígenas e não indígenas - Brasil, 2011 a 2022.

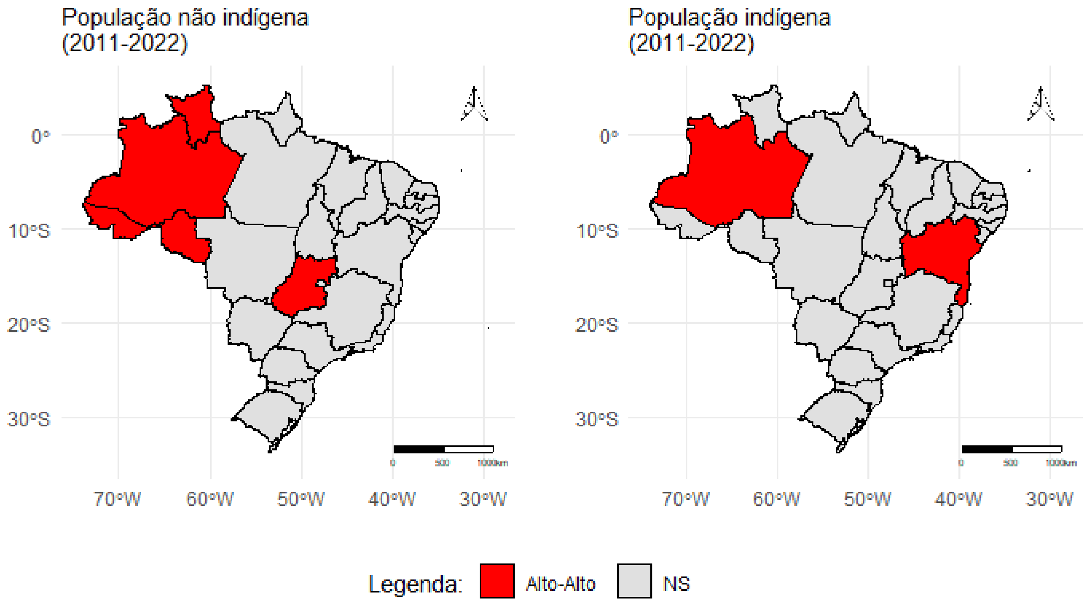

Material Suplementar 2 – Tabela de número de casos novos de TB e população indígena - 2011 a 2017

Região	UF	Ano													
		2011		2012		2013		2014		2015		2016		2017	
		Casos	População	Casos	População	Casos	População	Casos	População	Casos	População	Casos	População	Casos	População
**Centro-Oeste**		**73**	**133823,6**	**202**	**137389,4**	**211**	**141114,9**	**210**	**145003,5**	**308**	**149059,1**	**296**	**153285,5**	**191**	**157687,2**
	Distrito Federal	1	7061,654	0	7053,319	2	7044,993	2	7036,677	4	7028,371	2	7020,074	0	7011,788
	Goiás	2	7575,316	3	7158,86	1	6765,298	4	6393,372	0	6041,893	7	5709,737	2	5395,842
	Mato Grosso	70	44955,95	84	46755,14	80	48626,34	115	50572,42	234	52596,39	186	54701,35	81	56890,56
	Mato Grosso do Sul	183	74230,63	115	76422,11	128	78678,28	89	81001,07	70	83392,42	101	85854,38	108	88389,01
**Nordeste**		**51**	**214761**	**89**	**220369,1**	**112**	**226296,1**	**123**	**232557,8**	**102**	**239170,5**	**102**	**246151,8**	**96**	**253520**
	Alagoas	8	14359,9	1	14971,82	8	15609,81	6	16274,98	3	16968,5	5	17691,57	5	18445,46
	Bahia	22	56042,12	24	55350,87	20	54668,15	20	53993,85	15	53327,86	17	52670,1	11	52020,44
	Ceará	21	20998,75	12	22061,72	9	23178,5	17	24351,81	20	25584,51	15	26879,61	12	28240,27
	Maranhão	40	35101,13	27	35880,18	43	36676,53	43	37490,54	39	38322,62	42	39173,17	46	40042,6
	Paraíba	4	20816,73	5	22233,77	2	23747,28	4	25363,81	1	27090,39	0	28934,49	1	30904,13
	Pernambuco	18	56049,04	12	58550,99	22	61164,63	23	63894,93	17	66747,12	18	69726,61	17	72839,11
	Piauí	5	3441,154	2	3522,171	5	3605,095	6	3689,972	3	3776,848	1	3865,768	1	3956,782
	Rio Grande do Norte	5	2752,603	4	2717,655	3	2683,151	2	2649,084	4	2615,451	1	2582,244	2	2549,459
	Sergipe	3	5199,539	2	5079,895	0	4963,005	2	4848,805	0	4737,232	3	4628,226	1	4521,729
**Norte**		**144**	**316803,8**	**281**	**328999,5**	**293**	**341766,5**	**280**	**355133,5**	**275**	**369130,9**	**265**	**383790,6**	**337**	**399146,1**
	Acre	23	16797,84	18	17967,87	21	19219,4	24	20558,11	18	21990,05	19	23521,74	26	25160,12
	Amapá	4	7636,121	3	7939,862	9	8255,685	2	8584,071	7	8925,518	1	9280,547	3	9649,699
	Amazonas	117	173731,7	132	180602,9	162	187745,8	166	195171,2	144	202890,2	144	210914,6	173	219256,3
	Pará	40	38179,6	62	38225,25	39	38270,96	33	38316,72	45	38362,54	55	38408,41	73	38454,34

	Rondônia	29	13954,86	25	14297,95	25	14649,48	19	15009,65	24	15378,68	15	15756,78	16	16144,17
	Roraima	45	53409,38	31	56615,29	34	60013,64	30	63615,98	31	67434,55	22	71482,33	35	75773,08
	Tocantins	4	13094,24	10	13350,4	3	13611,58	6	13877,86	6	14149,35	9	14426,15	11	14708,36
**Sudeste**		**36**	**96883,43**	**125**	**92691,74**	**129**	**88708**	**93**	**84920,17**	**97**	**81316,99**	**96**	**77887,94**	**67**	**74623,21**
	Espírito Santo	3	9371,274	3	9112,876	3	8861,602	2	8617,257	4	8379,649	1	8148,593	0	7923,908
	Minas Gerais	8	30262,18	8	28980,08	17	27752,29	12	26576,53	5	25450,58	7	24372,32	10	23339,76
	Rio de Janeiro	25	14005,44	18	12855,71	25	11800,36	15	10831,65	19	9942,455	21	9126,26	17	8377,068
	São Paulo	143	43244,54	96	41743,08	84	40293,75	64	38894,74	69	37544,31	67	36240,76	40	34982,48
**Sul**		**31**	**74342,15**	**32**	**73522,25**	**32**	**72722**	**27**	**71941,12**	**22**	**71179,31**	**32**	**70436,29**	**47**	**69711,8**
	Paraná	6	25277,05	10	24777,19	5	24287,21	11	23806,91	9	23336,12	9	22874,64	7	22422,28
	Rio Grande do Sul	22	32642,53	15	32139,92	22	31645,05	14	31157,8	9	30678,05	14	30205,69	33	29740,6
	Santa Catarina	3	16422,57	7	16605,14	5	16789,75	2	16976,4	4	17165,13	9	17355,96	7	17548,91
**Brasil**		**335**	**836613,9**	**729**	**852972**	**777**	**870607,6**	**733**	**889556,2**	**804**	**909856,8**	**791**	**931552,1**	**738**	**954688,3**

Material Suplementar 3 – Tabela de número de casos novos de TB e população indígena - 2018 a 2022.

Região	UF	Ano											
		2018		2019		2020		2021		2022		Total	
		Casos	População	Casos	População	Casos	População	Casos	População	Casos	População	Casos	População
**Centro-Oeste**		**207**	**162268,6**	**195**	**167034,4**	**150**	**171989,8**	**110**	**177139,9**	**193**	**182491**	**2346**	**1878287**
	Distrito Federal	1	7003,511	2	6995,244	0	6986,986	3	6978,739	6	6971	23	84192,35
	Goiás	2	5099,203	1	4818,871	3	4553,952	2	4303,596	6	4067	33	67882,94
	Mato Grosso	76	59167,39	91	61535,34	72	63998,05	45	66559,33	70	69223	1204	675581,3
	Mato Grosso do Sul	128	90998,48	101	93684,99	75	96450,81	60	99298,28	111	102230	1269	1050630
**Nordeste**		**94**	**261294,5**	**110**	**269495,9**	**91**	**278145,7**	**56**	**287266,9**	**120**	**296884**	**1146**	**3025913**
	Alagoas	6	19231,46	0	20050,97	7	20905,39	5	21796,23	6	22725	60	219031,1
	Bahia	19	51378,8	25	50745,07	21	50119,16	6	49500,96	28	48890	228	628707,4
	Ceará	15	29669,81	14	31171,72	8	32749,64	7	34407,45	12	36149	162	335442,8
	Maranhão	25	40931,32	44	41839,77	28	42768,38	19	43717,6	34	44688	430	476631,8
	Paraíba	4	33007,85	1	35254,77	1	37654,65	2	40217,89	10	42956	35	368181,8
	Pernambuco	22	76090,55	18	79487,13	20	83035,33	12	86741,91	25	90614	224	864941,4
	Piauí	1	4049,939	4	4145,289	2	4242,884	1	4342,777	1	4445	32	47083,68
	Rio Grande do Norte	1	2517,091	1	2485,133	3	2453,581	2	2422,429	2	2392	30	30819,88
	Sergipe	1	4417,682	3	4316,03	1	4216,716	2	4119,688	2	4025	20	55073,55
**Norte**		**339**	**415233**	**354**	**432088,7**	**281**	**449752,6**	**221**	**468266,1**	**341**	**487674**	**3411**	**4747785**
	Acre	19	26912,61	9	28787,18	14	30792,31	18	32937,11	31	35231	240	299875,3
	Amapá	5	10033,53	10	10432,64	4	10847,61	1	11279,1	7	11728	56	114592,4
	Amazonas	171	227928	166	236942,6	152	246313,8	93	256055,5	149	266183	1769	2603736
	Pará	53	38500,32	83	38546,35	50	38592,45	48	38638,59	73	38685	654	461180,5
	Rondônia	15	16541,09	11	16947,77	11	17364,45	5	17791,37	14	18229	209	192065,3

	Roraima	66	80321,39	66	85142,7	38	90253,42	49	95670,91	63	101414	510	901146,7
	Tocantins	10	14996,1	9	15289,46	12	15588,57	7	15893,52	4	16204	91	175189,6
**Sudeste**		**68**	**71513,61**	**51**	**68550,55**	**50**	**65726**	**17**	**63032,42**	**51**	**60463**	**880**	**926317,1**
	Espírito Santo	5	7705,418	1	7492,953	0	7286,346	0	7085,436	0	6890	22	96875,31
	Minas Gerais	5	22350,93	6	21404	10	20497,19	7	19628,8	6	18797	101	289411,7
	Rio de Janeiro	13	7689,379	20	7058,143	15	6478,727	10	5946,876	12	5459	210	109571,1
	São Paulo	45	33767,88	24	32595,45	25	31463,73	0	30371,31	33	29317	690	430459
**Sul**		**20**	**69005,56**	**37**	**68317,31**	**21**	**67646,78**	**10**	**66993,74**	**37**	**66357**	**348**	**842175,3**
	Paraná	9	21978,87	14	21544,23	4	21118,18	4	20700,56	7	20291	95	272414,3
	Rio Grande do Sul	7	29282,68	18	28831,8	11	28387,87	4	27950,77	24	27520	193	360182,8
	Santa Catarina	4	17744,01	5	17941,28	6	18140,73	2	18342,41	6	18546	60	209578,3
**Brasil**		**728**	**979315,3**	**747**	**1005487**	**593**	**1033261**	**414**	**1062699**	**742**	**1093869**	**8131**	**11420478**

Material Suplementar 4 – Tabela de número de casos novos de TB e população não indígena - 2011 a 2017.

Região	UF	Ano													
		2011		2012		2013		2014		2015		2016		2017	
		Casos	População	Casos	População	Casos	População	Casos	População	Casos	População	Casos	População	Casos	População
**Centro-Oeste**		**2268**	**14199995,96**	**2985**	**14480306,39**	**3202**	**14766207,75**	**3014**	**15057812,74**	**2696**	**15355236,36**	**2699**	**15658595,95**	**2804**	**15968011,27**
	Distrito Federal	422	2622213,774	430	2683595,529	386	2746414,132	398	2810703,216	384	2876497,202	347	2943831,319	291	3012741,618
	Goiás	808	6110933,998	767	6228688,153	802	6348711,362	774	6471047,349	863	6595740,679	782	6722836,778	852	6852381,945
	Mato Grosso	1038	3050113,863	1147	3110683,092	1368	3172455,105	1270	3235453,789	842	3299703,502	902	3365229,087	1018	3432055,88
	Mato Grosso do Sul	705	2416734,327	641	2457339,619	646	2498627,15	572	2540608,383	607	2583294,972	668	2626698,77	643	2670831,825
**Nordeste**		**9461**	**53469187,79**	**16777**	**54080431,2**	**16841**	**54699092,9**	**15927**	**55325267,83**	**15302**	**55959052,18**	**15560**	**56600543,42**	**16949**	**57249840,35**
	Alagoas	1021	3140073,164	960	3173790,53	941	3207869,946	908	3242315,298	778	3277130,517	852	3312319,573	867	3347886,481
	Bahia	4914	14065357,1	3934	14176801,26	3978	14289128,42	3800	14402345,59	3552	14516459,82	3319	14631478,2	3759	14747407,92
	Ceará	3526	8547628,748	3191	8664604,724	3092	8783181,538	2911	8903381,099	3027	9025225,615	3100	9148737,598	3168	9273939,865
	Maranhão	1987	6644222,092	1666	6749761,398	1805	6856977,129	1650	6965895,914	1779	7076544,807	1839	7188951,288	1965	7303143,275
	Paraíba	1058	3782271,217	1040	3818326,9	1042	3854726,296	886	3891472,68	566	3928569,36	794	3966019,676	886	4003826,999
	Pernambuco	4058	8838879,116	3911	8936405,576	3726	9035008,124	3622	9134698,633	3653	9235489,108	3589	9337391,685	4023	9440418,635
	Piauí	830	3146703,458	733	3178731,625	735	3211085,785	658	3243769,256	573	3276785,39	627	3310137,573	664	3343829,225
	Rio Grande do Norte	952	3209062,25	858	3253860,263	920	3299283,65	879	3345341,14	794	3392041,585	843	3439393,962	989	3487407,37
	Sergipe	561	2094990,646	484	2128148,925	602	2161832,014	613	2196048,219	580	2230805,977	597	2266113,862	628	2301980,578
**Norte**		**2576**	**15895583,99**	**6450**	**16242993,99**	**6966**	**16598315,6**	**6859**	**16961736,96**	**6460**	**17333450,91**	**6921**	**17713655,12**	**7731**	**18102552,17**
	Acre	320	737756,1435	349	758356,9066	332	779532,916	370	801300,2345	313	823675,3736	352	846675,3059	414	870317,4776
	Amapá	225	685753,1182	199	710167,567	179	735451,2284	159	761635,0486	152	788751,0752	206	816832,4972	252	845913,6849
	Amazonas	2031	3390428,678	2125	3465683,457	2360	3542608,611	2388	3621241,214	2534	3701619,165	2478	3783781,205	2789	3867766,933
	Pará	3644	7705226,695	3019	7872387,866	3279	8043175,518	3159	8217668,326	2714	8395946,671	3043	8578092,678	3444	8764190,255

	Rondônia	535	1567709,99	526	1588797,876	538	1610169,424	531	1631828,449	489	1653778,818	574	1676024,45	532	1698569,317
	Roraima	111	412581,5874	87	425458,9327	119	438738,2011	98	452431,9371	113	466553,0769	113	481114,9607	155	496131,3446
	Tocantins	178	1396127,774	145	1422141,384	159	1448639,699	154	1475631,748	145	1503126,731	155	1531134,02	145	1559663,16
**Sudeste**		**16150**	**81138583,55**	**27793**	**82046085,93**	**27278**	**82963804,54**	**27228**	**83891855,11**	**27291**	**84830354,68**	**27696**	**85779421,63**	**30290**	**86739175,68**
	Espírito Santo	1234	3552040,871	1160	3599493,311	1027	3647579,679	984	3696308,441	998	3745688,182	930	3795727,596	1039	3846435,498
	Minas Gerais	3804	19753605,81	3104	19945831,18	3049	20139927,11	3116	20335911,83	2849	20533803,7	2921	20733621,28	3259	20935383,33
	Rio de Janeiro	11112	16154980,18	9423	16340799,41	9032	16528755,98	8906	16718874,48	8649	16911179,78	9104	17105697,04	9480	17302451,68
	São Paulo	16115	41677956,69	14106	42159962,03	14170	42647541,76	14222	43140760,35	14795	43639683,01	14741	44144375,71	16512	44654905,18
**Sul**		**4017**	**27564335,6**	**8200**	**27820764,93**	**8148**	**28080049,05**	**8067**	**28342224,53**	**7897**	**28607328,45**	**7622**	**28875398,41**	**8050**	**29146472,56**
	Paraná	2289	10515712,5	2075	10613901,35	2150	10713007,02	2078	10813038,07	2016	10914003,15	2039	11015910,97	1943	11118770,35
	Rio Grande do Sul	6	10716616,89	4395	10772869,51	4291	10829417,4	4241	10886262,12	4221	10943405,23	3908	11000848,29	4329	11058592,87
	Santa Catarina	1722	6332006,212	1730	6433994,075	1707	6537624,629	1748	6642924,331	1660	6749920,066	1675	6858639,151	1778	6969109,345
**Brasil**		**34472**	**192267686,9**	**62205**	**194670582,4**	**62435**	**197107469,8**	**61095**	**199578897,2**	**59646**	**202085422,6**	**60498**	**204627614,5**	**65824**	**207206052**

Material Suplementar 5 – Tabela de número de casos novos de TB e população não indígena - 2018 a 2022.

Região	UF	Ano											
		2018		2019		2020		2021		2022		Total	
		Casos	População	Casos	População	Casos	População	Casos	População	Casos	População	Casos	População
**Centro-Oeste**		**3165**	**16283604,48**	**3229**	**16605500,26**	**2722**	**16933825,83**	**2064**	**17268710,99**	**3501**	**17610289**	**34349**	**190188097**
	Distrito Federal	338	3083264,995	321	3155439,209	247	3229302,904	168	3304895,628	316	3382258	4048	35851157,53
	Goiás	908	6984423,372	852	7119009,161	783	7256188,34	635	7396010,882	909	7538528	9735	81624500,02
	Mato Grosso	977	3500209,722	1115	3569716,964	902	3640604,482	686	3712899,687	1036	3786631	12301	40875756,17
	Mato Grosso do Sul	942	2715706,393	941	2761334,93	790	2807730,105	575	2854904,798	1240	2902872	8970	31836683,27
**Nordeste**		**16203**	**57907043,06**	**16708**	**58572253,03**	**14105**	**59245573,04**	**12334**	**59927107,29**	**18778**	**60616960**	**184945**	**683652352,1**
	Alagoas	842	3383835,298	806	3420170,124	670	3456895,106	569	3494014,432	845	3531532	10059	39987832,47
	Bahia	2815	14864256,18	3503	14982030,26	2639	15100737,51	2287	15220385,31	3851	15340981	42351	176337368,6
	Ceará	3291	9400855,551	3073	9529508,102	2600	9659921,289	2025	9792119,206	3161	9926126	36165	110655229,3
	Maranhão	2053	7419149,131	2045	7536997,667	1857	7656718,153	1857	7778340,324	2365	7901894	22868	87078595,18
	Paraíba	964	4041994,732	964	4080526,311	790	4119425,205	669	4158694,914	1097	4198339	10756	47844193,29
	Pernambuco	3746	9544582,364	3930	9649895,415	3216	9756370,47	3042	9864020,35	4620	9972858	45136	112746017,5
	Piauí	652	3377863,802	620	3412244,794	537	3446975,726	542	3482060,161	694	3517502	7865	39947688,8
	Rio Grande do Norte	1108	3536091,038	989	3585454,322	1135	3635506,711	799	3686257,823	1265	3737717	11531	41607417,11
	Sergipe	732	2338414,972	778	2375426,028	661	2413022,873	544	2451214,779	880	2490011	7660	27448009,87
**Norte**		**7833**	**18500349,75**	**8781**	**18907260,71**	**7561**	**19323503,28**	**6541**	**19749301,15**	**9767**	**20184883**	**84446**	**215513586,6**
	Acre	394	894619,8224	505	919600,7748	496	945279,2838	434	971674,8276	486	998807	4765	10347596,07
	Amapá	218	876030,2323	277	907219,0008	242	939518,1638	268	972967,2542	395	1007607	2772	10047845,87
	Amazonas	2808	3953616,829	2922	4041372,27	2510	4131075,553	2511	4222769,911	3359	4316500	30815	46038463,83
	Pará	3564	8954325,13	4101	9148584,888	3502	9347059,018	2577	9549838,949	4463	9757018	40509	104333514

	Rondônia	508	1721417,444	571	1744572,91	420	1768039,85	346	1791822,452	530	1815925	6100	20268655,98
	Roraima	164	511616,4143	224	527584,7984	239	544051,5819	251	561032,3206	359	578543	2033	5895838,156
	Tocantins	177	1588723,875	181	1618326,068	152	1648479,83	154	1679195,438	175	1710483	1920	18581672,73
**Sudeste**		**30594**	**87709737,93**	**30184**	**88691230,84**	**25346**	**89683778,28**	**9749**	**90687505,53**	**34897**	**91702539**	**314496**	**1035864073**
	Espírito Santo	1099	3897820,816	1042	3949892,602	107	4002660,025	31	4056132,379	1294	4110319	10945	45900098,4
	Minas Gerais	3217	21139108,75	3312	21344816,65	2864	21552526,33	2237	21762257,26	3410	21974029	37142	250150822,2
	Rio de Janeiro	10292	17501469,45	10080	17702776,39	8604	17906398,81	7394	18112363,35	11971	18320697	114047	206606443,6
	São Paulo	15986	45171338,92	15750	45693745,21	13771	46222193,13	87	46756752,54	18222	47297494	168477	533206708,5
**Sul**		**7967**	**29420589,56**	**8091**	**29697788,63**	**6798**	**29978109,54**	**5842**	**30261592,63**	**8129**	**30548278**	**88828**	**348342931,9**
	Paraná	2166	11222590,15	2133	11327379,36	1871	11433147,02	1366	11539902,27	2035	11647654	24161	132875016,2
	Rio Grande do Sul	4195	11116640,55	4349	11174992,94	3690	11233651,62	3317	11292618,21	4417	11351894	45359	132377809,6
	Santa Catarina	1606	7081358,851	1609	7195416,329	1237	7311310,899	1159	7429072,15	1677	7548730	19308	83090106,04
**Brasil**		**65762**	**209821324,8**	**66993**	**212474033,5**	**56532**	**215164790**	**36530**	**217894217,6**	**75072**	**220662949**	**707064**	**2473561040**
