# Supplementary material for: Spatial-temporal evolution of tuberculosis incidence rates in indigenous and non-indigenous people of Brazil, from 2011 to 2022
Source: Rev Bras Epidemiol. 2023 Dec 11;26:e230055. doi: 10.1590/1980-549720230055 (PMC10715319; doi:10.1590/1980-549720230055)
Supplement: Supplementary file 2 [file 1980-5497-rbepid-26-e230055-s2.docx]

**Tabela 1** – Características clínicas e sociodemográficas da amostra estratificada por sexo ELSA-Brasil (2008-2010).

|  | **Homens** | **Mulheres** | **All** |
| --- | --- | --- | --- |
| **Geral**  N(%) | 5984 (46,7) | 6829 (53,3) | 12813 (100,0) |
| Idade (anos)  Altura (cm)  Peso (kg) | 52 ± 9 172 ± 7 79,7 ± 13,8 | 52 ±9 159 ± 6 67,5 ± 12,8 | 52 ± 9 165 ± 9 73,3 ± 14,6 |
| **Raça/Cor** |  |  |  |
| Brancos | 3150 (52,6) | 3564 (52,2) | 6714 (52,4) |
| Pretos | 805 (13,5) | 1168 (17,1) | 1973 (15,4) |
| Pardos | 1735 (29,0) | 1775 (26,0) | 3510 (27,4) |
| Outros | 354 (4,9) | 322 (4,7) | 676 (3,6) |
| **Escolaridade** |  |  |  |
| <4 anos | 398 (7,3) | 260 (3,8) | 658 (5,2) |
| 4 - 8 anos | 491 (8,2) | 355 (5,2) | 846 (6,7) |
| 9 - 11 anos | 1932 (32,3) | 2424 (35,5) | 4356 (34,2) |
| ...≥12 anos | 3123 (52,2) | 3790 (55,5) | 6913 (53,9) |
|  |  |  |  |
| **Parâmetros** |  |  |  |
| IMC (kg/m^2^) | 26,67 ± 4,07 | 26,67 ± 4,71 | 26,8 ± 4,42 |
| PAS(mmHg) | 131 ± 17 | 122 ± 17 | 126,6 ± 17,6 |
| PAD(mmHg) | 79 ± 10 | 74 ± 10 | 76 ± 10 |
| Glicemia (mg/dL) | 117 ± 34 | 107 ± 26 | 112 ± 31 |
| Colesterol (mg/dL) | 212 ± 44 | 217 ± 42 | 215,0 ± 42,7 |
| Triglicerídeos (mg/dL) | 162 ± 130 | 119 ± 83 | 139,7 ± 109,6 |
| Creatinina sérica (mg/dL) | 1,10 ± 0,20 | 0,82 ± 0,13 | 0,94 ± 0,21 |
| **Subcategorias** |  |  |  |
| Peso Normal (<25kg/m^2^) | 2003 (33,5) | 2725 (39,9) | 4728 (36,9) |
| Sobrepeso(25.0-29.9kg/m^2^) | 2796 (46,7) | 2534 (37,1) | 5330 (41,6) |
| Obesidade (≥30kg/m^2^) | 1130 (18,9) | 1509 (22,0) | 2639 (20,6) |
| Baixo Peso (<18,5kg/m^2^) | 55 (0,9) | 61 (1,0) | 116 (0,9) |
| Normotensos | 4036 (67,4) | 4280 (75,0) | 8316 (64,9) |
| Hipertensos | 2703 (32,6) | 1794 (25,0) | 4497 (35,1) |
| Diabéticos | 789 (13,2) | 505 (7,4) | 1294 (10,1) |

**Dados representam:** Número de indivíduos – N (percentagem) OU média ± desvio padrão;
IMC: índice de massa corporal; PAS: pressão arterial sistólica ;PAD: pressão arterial diastólica.


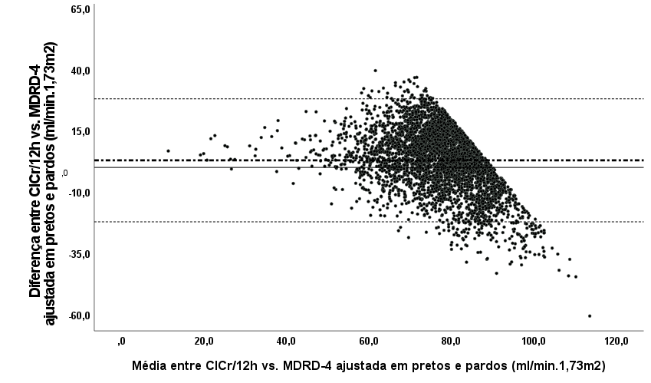

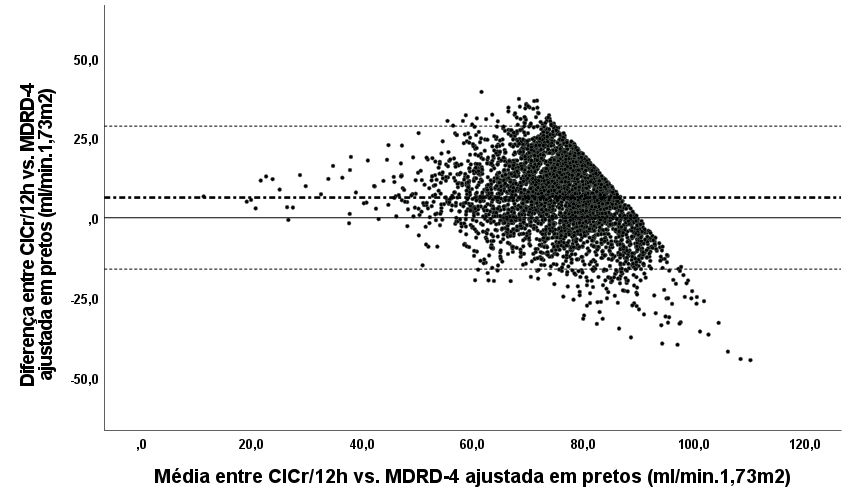
**Figura Suplementar–** Concordância entre a TFG medida em urina de 12h e estimada por MDRD-4 e por CKD-EPI sem ajustes com dados de CrCl<90 mL.min.1,73m^2^. ELSA-Brasil (2008-2010).

**(B)**

**B2**

**B1**

**B3**

**(A)**

**A2**

**A1**

**A3**

**Dif. entre ClCr/12h *e* MDRD-4**

**Dif. entre ClCr/12h *e* MDRD-4**

**IC=50,18**

**IC=44,80**

**Média entre ClCr/12h *e* MDRD-4**

**Média entre ClCr/12h *e* MDRD-4**


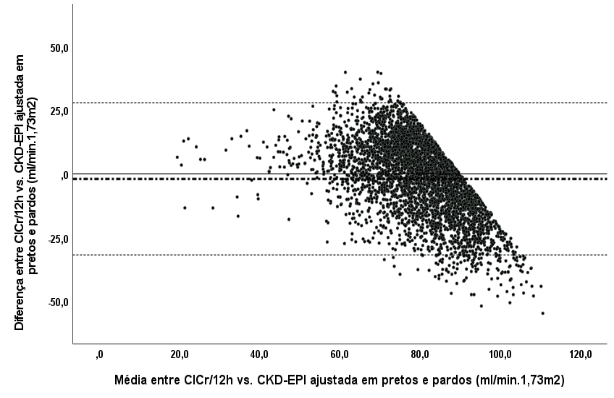

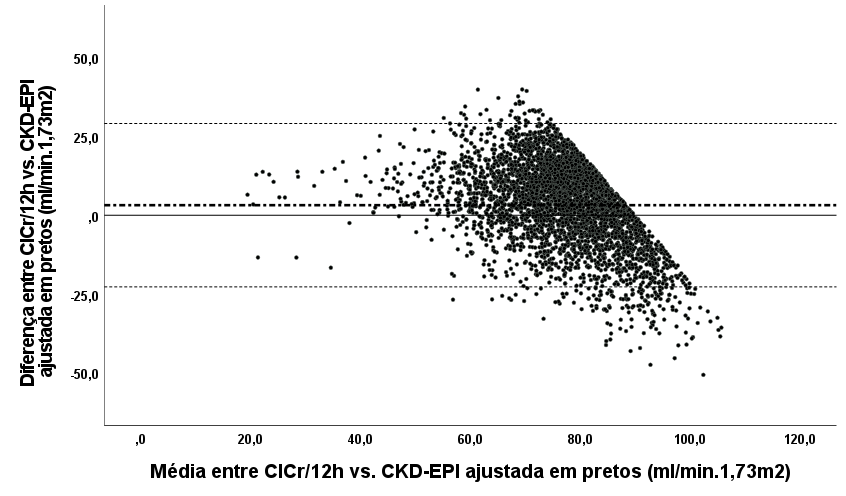


**(D)**

**D2**

**D1**

**D3**

**(C)


C2**

**C1**

**C3**

**Dif. entre ClCr/12h *e* CKDEPI**

**Dif. entre ClCr/12h *e* CKDEPI**

**IC=59,66**

**IC=51,78**

**Média entre ClCr/12h *e* CKDEPI**

**Média entre ClCr/12h *e* CKDEPI**

**Legenda:**  **A1, B1, C1, D1 -** Médias das diferenças

**A2, B2, C2, D2**: Limites superiores dos intervalos de concordância

**A3, B3, C3, D3**: Limites inferiores dos intervalos de concordância

**(A),(B)**  Ajuste em pretos; **(C),(D)** – Ajuste em pretos/pardos
